# Exposure to radial extracorporeal shock waves modulates viability and gene expression of human skeletal muscle cells: a controlled in vitro study

**DOI:** 10.1186/s13018-018-0779-0

**Published:** 2018-04-06

**Authors:** Stefan G. Mattyasovszky, Eva K. Langendorf, Ulrike Ritz, Christoph Schmitz, Irene Schmidtmann, Tobias E. Nowak, Daniel Wagner, Alexander Hofmann, Pol M. Rommens, Philipp Drees

**Affiliations:** 1 Department of Orthopedics and Traumatology, University Medical Centre of the Johannes Gutenberg-University of Mainz, Langenbeckstraße 1, 55131 Mainz, Germany; 2 Institue for Medical Biometry, Epidemiology and Computer Science, University Medical Centre of the Johannes Gutenberg-University of Mainz, Mainz, Germany; 3 Extracorporeal Shock Wave Research Unit, Department of Anatomy II, Ludwig-Maximilians-University of Munich, Munich, Germany

**Keywords:** Shock wave therapy, Muscle injury, Primary muscle cells

## Abstract

**Background:**

Recent clinical and animal studies have shown that extracorporeal shock wave therapy has a promoting influence on the healing process of musculoskeletal disorders. However, the underlying biological effects of extracorporeal shock wave therapy on human skeletal muscle cells have not yet been investigated.

**Methods:**

In this study, we investigated human skeletal muscle cells after exposure to radial extracorporeal shock waves in a standardized in vitro setup. Cells were isolated from muscle specimens taken from adult patients undergoing spine surgery. Primary muscle cells were exposed once or twice to radial extracorporeal shock waves in vitro with different energy flux densities. Cell viability and gene expression of the paired box protein 7 (Pax7), neural cell adhesion molecule (NCAM), and myogenic factor 5 (Myf5) and MyoD as muscle cell markers were compared to non-treated muscle cells that served as controls.

**Results:**

Isolated muscle cells were positive for the hallmark protein of satellite cells, Pax7, as well as for the muscle cell markers NCAM, MyoD, and Myf5. Exposure to radial extracorporeal shock waves at low energy flux densities enhanced cell viability, whereas higher energy flux densities had no further significant impact. Gene expression analyses of muscle specific genes (Pax7, NCAM, Myf5, and MyoD) demonstrated a significant increase after single exposure to the highest EFD (4 bar, 0.19 mJ/mm^2^) and after double exposure with the medium EFDs (2 and 3 bar; 0.09 and 0.14 mJ/mm^2^, respectively). Double exposure of the highest EFD, however, results in a significant down-regulation when compared to single exposure with this EFD.

**Conclusions:**

This is the first study demonstrating that radial extracorporal shock wave therapy has the potential to modulate the biological function of human skeletal muscle cells. Based on our experimental findings, we hypothesize that radial extracorporal shock wave therapy could be a promising therapeutic modality to improve the healing process of sports-related structural muscle injuries.

## Background

Muscle injuries are very common in athletes. They represent more than 30% of all injuries in professional soccer and cause about one quarter of total injury absence [1]. Despite the growth of knowledge in therapy in professional sports, the treatment options of structural muscle injuries are still very limited. Furthermore, there is no consensus on their management so far. Recently, several injection methods were reported to shorten the recovery time after muscle strain injuries [2]. The injection of local anesthetics, Actovegin and Traumeel, as well as platelet-rich plasma (PRP) were most frequently reported but the results concerning these therapies are ambivalent [3–5]. Despite the fact that there are few clinical studies illustrating successful therapy, only limited scientific evidence is available supporting the general use of these agents.

Extracorporeal shock wave therapy (ESWT) may serve as a non-invasive therapeutic option for muscle injuries. Two types of extracorporeal shock waves (ESWs) are used in medical therapy: focused extracorporeal shock waves (fESWs) and radial extracorporeal shock waves (rESWs) [6]. Both are single acoustic impulses with an initial high positive peak pressure between 10 and 100 megapascals (MPa) reached in less than 1 μs [7]. The positive pressure amplitude is followed by a low tensile amplitude of a few microseconds duration that can generate cavitation [8, 9]. They are further characterized by a short life cycle of approximately 10–20 μs and a broad frequency spectrum [10]. Focused ESWs differ from rESWs in the penetration depth into the tissue, some physical characteristics, and the technique for generating them [6, 9].

For more than a decade, ESWT has been successfully used in the treatment of sports-related over-use tendinopathies such as proximal plantar fasciopathy, Achilles tendinopathy, proximal hamstring tendinopathy, lateral epicondylitis of the elbow, and calcifying or non-calcific tendinitis of the shoulder [6, 11–15]. Besides this, ESWT can reduce pain and muscle tone in multiple sclerosis (MS) patients without adverse effects, and is a therapeutic option for treating pain and muscle hypertonia in patients with cerebral palsy and stroke [16]. It is effective for the purpose of pain relief and improving cervical range of motion in patients with myofascial pain syndrome [17]. Furthermore, ESWT can improve microcirculation blood flow of ischemic limbs in patients with peripheral arterial disease [18]. The primary advantage of ESWT is its non-invasive nature and very low rate of complications when applied to musculoskeletal tissues.

Human skeletal muscle tissue possesses a remarkable capacity to regenerate in response to muscle injury. Adult myofibers are terminally differentiated so that muscle repair is largely attributed to a distinct population of resident myogenic progenitor cells termed satellite cells [19]. This cell population is a pool of stem cells that reside between the sarcolemma and the basal lamina surrounding each myofiber in a quiescent state [20]. Upon muscle injury, satellite cells become activated, proliferate, and fuse either with existing fibers or with themselves to form new myofibers [19]. In immature skeletal muscle satellite cells, distinct patterns of myogenic proteins are expressed [20]. Quiescent satellite cells express the paired-box transcription factor paired box protein 7 (Pax7) [21] and when activated, they co-express Pax7 with myogenic differentiation (MyoD) [22], a key transcription factor for myogenic differentiation and a member of the myogenic regulatory factor (MRF) family, comprising MyoD, myogenic factor 5 (Myf5), Myogenin, and MRF-4 [23]. Pax7 is specifically required for maintenance of postnatal muscle. The lack of satellite cells in Pax7 knock-out mice suggests that this factor is critical for their biogenesis [24]. Most satellite cells proliferate, down-regulate Pax7, and differentiate. By contrast, others maintain Pax7 but lose MyoD and return to a state resembling quiescence [25]. Myf5 is a direct target gene of Pax7 and transcription of Myf5 directly varies with Pax7 levels [26]. Neural cell adhesion molecule (NCAM) is expressed in proliferating myoblasts among cells cultured from skeletal muscle [27]. Most insights into this complex network of muscle regeneration resulted from research on murine in vitro and in vivo models.

So far, little is known about the biological and molecular effects of ESWT on regenerative muscle cells. Based on the above-mentioned positive clinical and experimental findings, we hypothesize that radial extracorporeal shock wave therapy (rESWT) could be a promising therapeutic modality to improve the healing process of sports-related structural muscle injuries by regulating satellite cells. In the present study, for the first time, biological effects of rESWT on human primary skeletal muscle cells under controlled in vitro conditions are described.

## Methods

### Cell cultures

Human skeletal muscle cells were isolated from muscle specimens taken from four patients (all male, aged 27, 46, 55, and 67 years) undergoing spinal surgery for lumbar disc disease in two cases or fracture of the thoracic spine at our Department. Muscle tissue used in the present study was considered to be surgical waste and would otherwise have been discarded by the hospital. The patients included were neither operated before nor suffered from muscle diseases or any other condition known to affect muscle healing. All experiments were approved by the Ethics Committee of Landesärztekammer Rheinland—Pfalz by an arrangement concerning excess material. Written informed consent was obtained from every participating patient.

Muscle tissue was processed directly after excision. Cell isolation was performed according to an established protocol [27]. Fibrous tissue and perimysium were carefully removed under sterile conditions. The muscle tissue was cut into small pieces (1 mm^2^) and rinsed in phosphate-buffered saline (PBS, Dulbecco’s phosphate buffered saline, Gibco/Life Technologies, Paisley, UK) to remove blood and fat residues. After digestion with type II collagenase (Worthington Biochemical Corp., Lakewood, NJ, USA), muscle solution was treated with 0.25% trypsine/1 mM EDTA (Biochrom, Berlin, Germany) in a water bath at 37 °C. The specimens were filtered through a cell strainer (100 μm mesh; BD Biosciences, Heidelberg, Germany) to obtain a single-cell suspension and finally centrifuged at 1.4 × 10^3^ rpm. The pellet was re-suspended in medium (Skeletal Muscle Cell Growth Medium; PromoCell) supplemented with antibiotics (10,000 U/ml penicillin G sodium and 10,000 μg/ml streptomycin sulfate; Gibco/Life Technologies) and cells were seeded in collagen-coated (10% type I collagen; Discovery Labware, Woburn, MA, USA) culture flasks and incubated in a humidified atmosphere of 5% CO_2_ at 37 °C. Culture media were changed every second day, and preconfluent muscle cells were passaged using accutase (PAA Laboratories, Cölbe, Germany). For cell quantification, we used a Luna™ Cell Counting Slide and the Cell Counter Cellometer® Auto T4 (Nexcelom Bioscience LLC, Lawrence, MA 01843, USA). Early passage cells (passages 2–4) were used for all experiments. Cell differentiation was performed using the Skeletal Muscle Differentiation Medium (PromoCell, Heidelberg, Germany) by changing medium after cells reached 70–80% confluence for 5 days in culture. Due to limited material, not all experiments could be performed with cells isolated from all patients.

### Immunofluorescence staining

The phenotype of the cultured cells was determined by immunofluorescence staining. Cells were seeded on chamber slides (Chamber Slides System LAB-TEK, Nalgene Nunc International, Rochester, NY, USA) coated with collagen (10% type I collagen; Discovery Labware). Cells were fixed with − 20 °C methanol (AppliChem, Darmstadt, Germany) followed by washing in − 20 °C aceton (Sigma-Aldrich, St. Louis, MO, USA). After 20-min incubation in PBS, cells were incubated with primary antibodies against Pax7 (dilution 1:200, mouse monoclonal anti-PAX7 antibody, ab92317; Abcam, Cambridge, UK), NCAM (dilution 1:200, mouse monoclonal Anti-NCAM (ERIC-1), ab6123; Abcam), and Myf-5 (dilution 1:100, M-18 sc-31,949, Santa Cruz Biotechnology, Dallas, USA) in 0.5% BSA/PBS (PAA Laboratories, Pasching, Austria) overnight at 4 °C. Then cells were incubated with secondary antibodies anti-mouse Alexa Fluor™ 488 (dilution 1:200, A-11001, Invitrogen/Life Technologies, Carlsbad, CA, USA) and anti-goat Cy3 (dilution 1:450, 705-165-003, Dianova, Hamburg, Germany) in PBS containing 0.5% BSA for 2 h at room temperature in the dark and washed twice with PBS. Nuclei were stained with mounting medium containing DAPI (1.5 μg/ml, Vectashield, Vector Laboratories, Burlingame, CA, USA). Myosin antibody was used to detect myotube formation of differentiated skeletal muscle cells in our isolated cultures (dilution 1:200, mouse monoclonal Anti-Fast Myosin Skeletal Heavy chain antibody (MY-32), ab51263, Abcam). Secondary antibody (anti-mouse Alexa Fluor™ 488, A-11001, Invitrogen/Life Technologies) was used as described above.

### Microscopy

Microscopy was performed with an EVOS Digital Inverted Microscope (Life Technologies) and × 4 (Figs. 2a and 3j–l), × 10 (Fig. 2b), and × 40 (Fig. 3a–i) objectives. Both phase contrast and fluorescence modes were used. The final figures were assembled using Corel Photo-Paint X6 and Corel Draw X6 (both versions 16.1.0.843; Corel, Ottawa, Canada).

### Exposure of cells to radial extracorporeal shock waves in vitro

**Fig. 1:**
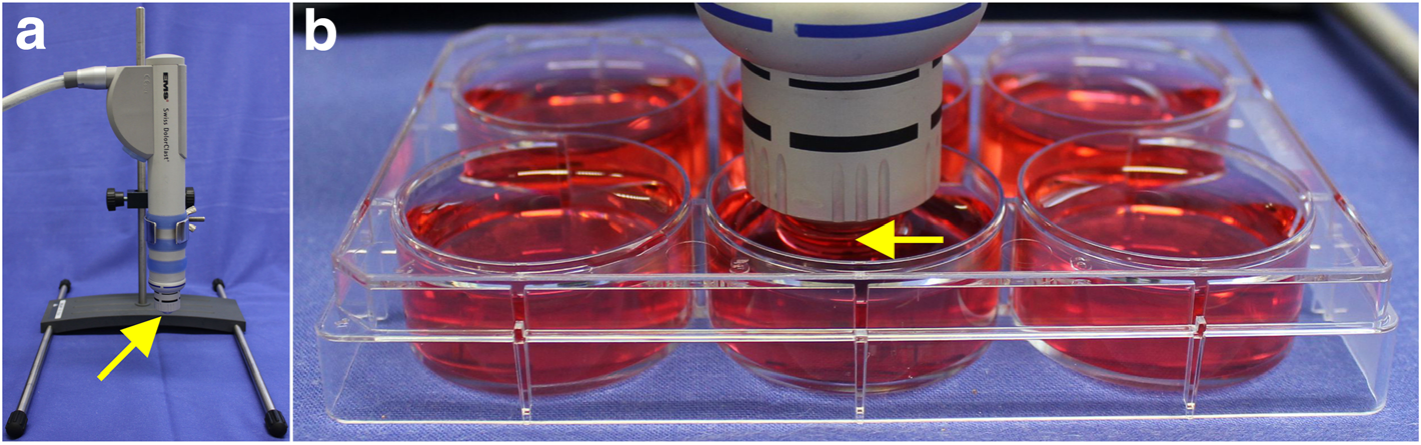
Exposure of human skeletal muscle cells to radial extracorporeal shock waves in vitro. **a** Positioning of the handpiece of a radial extracorporeal shock wave (rESW) device (Swiss DolorClast; Electro Medical Systems, Nyon, Switzerland) in a fixed position using a customized holder. **b** Exposure of cells cultivated at the bottom of 6-well plates to rESWs at a constant distance of 13 mm. The arrows point to the applicator of the handpiece that is emitting the rESWs

Exposure to radial extracorporeal shock waves (rESWs) in vitro was performed with a Swiss DolorClast device (Electro Medical Systems, Nyon, Switzerland) and its “Radial” handpiece (Fig. 1a). The rational for changing the pressure as well as the energy flux density directly reflected the working mechanism of the pneumatic radial extracorporeal shock wave device that was used in the present study (Swiss DolorClast, Electro Medical Systems, Nyon, Switzerland) and published by Császár et al. [8].

Both the 6-mm and the 10-mm applicators were used. Further, 8 × 10^4^ human muscle cells per well were seeded in 6-well plates and incubated for 2 days. Then 10 ml growth medium were added to each well, the “Radial” handpiece of the rESWT device was placed in a fixed position using a customized holder (Fig. 1a) and the applicator of the handpiece immerged into the solution with a distance of 13 mm to the bottom of the wells (Fig. 1b). The optimum number of cells per well and a suitable distance of the applicator to the bottom of the wells were determined in pre-tests (data not shown). Parameters were chosen according to the rate of viable cells after exposure to rESWs.

In the main experiments, rESWs were applied at a frequency of 10 per second (i.e., at 10 Hz) and 500 impulses. Different treatment of various groups is described in Table 1.

**Table 1:** Exposure of human skeletal muscle cells to radial extracorporeal shock waves (rESWs) in vitro

Group	Applicator [∅]	AP [bar/energy]	Energy flux density [%]	ESW-application on day 1	ESW-application on day 5
A0	–	–	–	–	–
A1	6 mm	1/0.05 mJ/mm^2^	5	+	–
A2	6 mm	2/0.09 mJ/mm^2^	10	+	–
A3	10 mm	3/0.14 mJ/mm^2^	75	+	–
A4	10 mm	4/0.19 mJ/mm^2^	100	+	–
B0	–	–	–	–	
B1	6 mm	1/0.05 mJ/mm^2^	5	+	+
B2	6 mm	2/0.09 mJ/mm^2^	10	+	+
B3	10 mm	3/0.14 mJ/mm^2^	75	+	+
B4	10 mm	4/0.19 mJ/mm^2^	100	+	+

AP, air pressure at which the rESW was operated; energy flux density of the rESWs applied on days 1 and day 5, groups A1–A4 were treated once, whereas groups B1–B4 were treated twice. A0 and B0 were used as untreated controls

Cells in groups A1 to A4 were exposed to rESWs on day 1 and were analyzed on day 3 (i.e., single exposure to rESWs), whereas cells in groups B1 to B4 were exposed to rESWs on days 1 and 5 and were analyzed on day 7. Energy flux density (EFD) data at a distance of 13 mm to the applicator were provided by Electro Medical Systems and were as follows: groups A1 and B1: 0.0006 mJ/mm^2^ (6-mm applicator; device operated at 1 bar air pressure; 5% of the EFD applied to groups A4 and B4); groups A2 and B2: 0.0012 mJ/mm^2^ (6-mm applicator; device operated at 2 bar air pressure; 10% of the EFD applied to groups A4 and B4); groups A3 and B3: 0.009 mJ/mm^2^ (10-mm applicator; device operated at 3 bar air pressure; 75% of the EFD applied to groups A4 and B4); and groups A4 and B4: 0.012 mJ/mm^2^ (10-mm applicator; device operated at 4 bar air pressure).

### Cell viability assay

Optimal number of cells was determined in pre-tests.

Viability assays were performed either on day 3 (single exposure to rESWs on day 1, groups A1 to A4) or day 7 (double exposure to rESWs on days 1 and 5, groups B1 to B4), respectively, using the alamarBlue® assay (Invitrogen/Life Technologies, Carlsbad, CA, USA) according to the manufacturer’s instructions. For every measurement, alamarBlue® solution was added to each well and incubated for 4 h. Samples were examined in triplicates, and the emission was measured at a wavelength of 580–640 nm using the GloMax® Multidetection System (Promega, Madison, WI, USA).

This experiment was performed with cells isolated from three patients and each cell type was used in triplicates. As we used a non-treated control as control for each treatment group, *n* for both control groups (A0 and B0) *n* = 36 and for all treated groups (A1–4 and B1–4) *n* = 9.

### Quantitative real-time polymerase chain reaction

Total RNA was extracted from cells on day 3 (groups A0 to A4) or day 7 (groups B0 to B4). The purification was performed using the RNeasy Mini Kit (QIAGEN, Hilden, Germany) according to the manufacturer’s instructions. RNA quality and concentration were measured photometrically. The A 260/280 ratio of samples was between 1.9 and 2.1. To avoid any contamination with DNA, DNase (QIAGEN) was used. Reverse transcription was performed using 2 μg RNA, Superscript-Reverse transcriptase III (Invitrogen/Life Technologies) Random Primers (Promega, Madison, WI, USA), and dNTPs (Bioron GmbH, Ludwigshafen, Germany).

Gene expression analyses for Pax7, NCAM, Myf5, and MyoD were performed by quantitative RT-PCR with QuantiTect Primer Assays (Table 2) (QIAGEN), the Power SYBR Green PCR Master Mix, and cDNA using the 7300 Real-Time PCR System (Applied Biosystems, Foster City, CA, USA).

**Table 2:** Primers used for quantitative real-time PCR in the present study (all primers from QIAGEN, Hilden, Germany)

Product name	Catalogue no.
Hs_MYF5_1_SG QuantiTect Primer Assay	QT00027825
Hs_NCAM1_1_SG QuantiTect Primer Assay	QT00071211
Hs_PAX7_1_SG QuantiTect Primer Assay	QT00018942
Hs_RRN18S_1_SG QuantiTect Primer Assay	QT00199367
Hs_Myod1_1_SG QuantiTect Primer Assay	QT00209713

All samples were examined in triplicates. Following steps were used for amplification: initial denaturation at 95 °C for 10 min, followed by 40 cycles of DNA amplification (denaturing for 15 s at 95 °C, annealing for 30 s at 55 °C, and elongation for 35 s at 72 °C) followed by dissociation curve. Gene expression was normalized using 18S. The comparative threshold cycle (Ct) method was used to calculate the expression and presented as 2-^ΔΔCt^ values, respectively.

This experiment was performed with cells isolated from four patients and each cell type was used in triplicates. As we used a non-treated control as control for each treatment group, *n* for both control groups (A0 and B0) *n* = 36. Due to limited material, cells from two patients were used for the treated groups (A1–4 and B1–4) resulting in *n* = 6.

### Statistical analysis

Mean and standard deviation were calculated for all variables of the cell viability assay. Effects of rESWs were tested using the *F* test followed by comparison of individual groups to the corresponding control group using Dunnett’s test. Furthermore, median were calculated for effects of rESWs on gene expression. Kruskal-Wallis test followed by comparison of individual groups to the corresponding control group and to each other group using the Mann-Whitney *U* test were used. For all analyses, a *p* value < 0.05 was considered statistically significant. Calculations were performed using SPSS (Version 23 for Windows; IBM, Armonk, NY, USA).

## Results

### Confirmation of the presence of muscle cells in primary culture

**Fig. 2:**
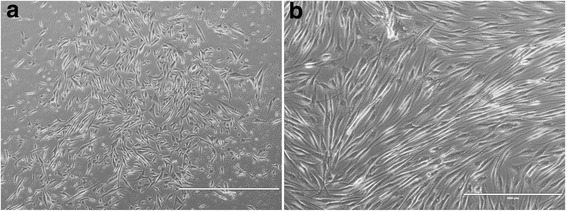
Low-resolution phase contrast of primary cultured human skeletal muscle cells and mature myotubes. **a**, **b** Phase contrast imaging of muscle cells; scale bar in (**a**) represents 1000 μm and 400 μm in (**b**)

Only a few days after seeding, cultured human skeletal muscle cells demonstrate the typical structure and formation of stellate cells (Fig. 2a). After 5 days in differentiation medium, cells were differentiated and started to form myotubes (Fig. 2b).

**Fig. 3:**
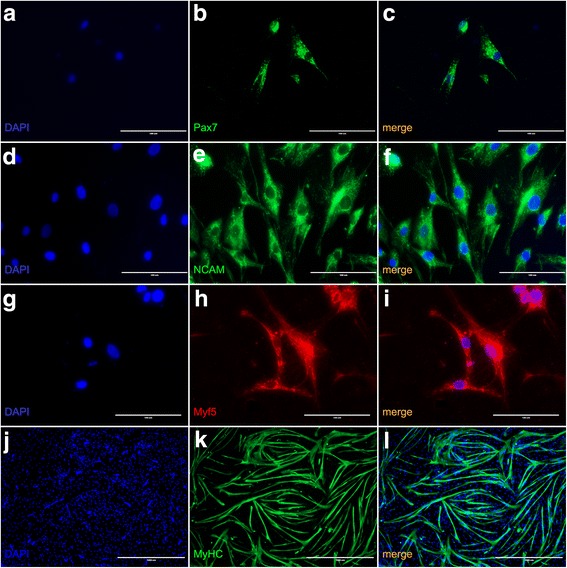
Immunofluorescence imaging of cultured human skeletal muscle cells. Immunofluorescence staining for Pax7 (**a**–**c**), NCAM (**d**–**f**), Myf5 (**g**–**i**), and Myosin (**j**–**l**). The scale bars represent 100 μm for **a**–**i** and 1000 μm for **j**–**l**.

Human skeletal muscle cell cultures were positive for Pax7 (Fig. 3a–c), NCAM (Fig. 3d–f), and Myf5 (Fig. 3g–i) demonstrated by immunofluorescence staining indicating the presence of a mixed muscle cell culture. Myosin staining (Fig. 3j–l) evidenced the presence of myotube formation of mature muscle cells. The percentage of Pax7 positive cells hallmarking satellite cells was comparatively low as expected.

### Viability of human skeletal muscle cells was influenced by exposure to radial extracorporeal shock waves in vitro in a dose-dependent manner

**Fig. 4:**
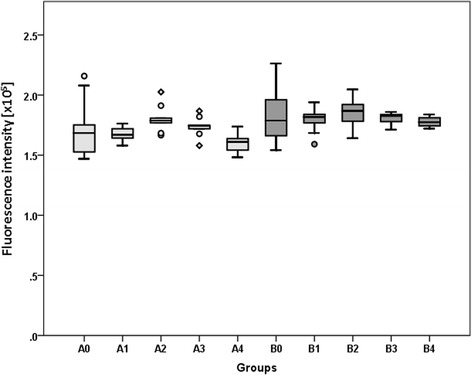
Results of alamarBlue® Assay to determine cell viability. The panels show Tukey boxplots of absolute values of the emission at 570 nm of the alamarBlue® assay performed for cells in groups A0 to A4 (light gray bars) and groups B0 to B4 (dark gray bars) after exposure to radial extracorporeal shock waves (groups A1–A4 and B1–B4) or sham-exposure (groups A0 and B0) as outlined in Table 1. Results of statistical analysis are indicated (Dunnett’s test; comparison to control)

Both single exposure as well as double exposure of human primary skeletal muscle cells to rESWs in vitro influenced viability of the cells. Medium strength of shock wave application (2 and 3 bar; 0.09 and 0.14 mJ/mm^2^) demonstrated the highest viability of muscle cells. Higher shock wave application resulted in low viability of the cells (Fig. 4). Although a tendency is clearly visible, the differences are not statistically significant.

### Exposure of human skeletal muscle cells to radial extracorporeal shock waves in vitro had a dose-dependent impact on gene expression

**Fig. 5:**
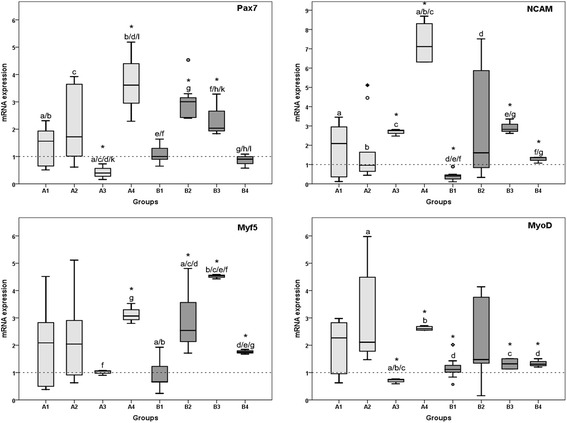
Results of qRT-PCR analysis. The panels show Tukey boxplots of 2^-ΔΔCt^ values of mRNA expression of Pax7, NCAM, Myf5, and MyoD determined with quantitative real-time polymerase chain reaction on day 3 (groups A1 to A4; light gray bars, single exposure to rESW) or day 7 (groups B1 to B4; dark gray bars, double exposure to rESW) compared to the non-treated groups (A0 and B0, respectively). Statistical significances between the groups on day 3 and day 7 as well as between day 3 and 7 are indicated with same letters. Statistical significance to the control groups are indicated with asterisks (Mann-Whitney *U* test; comparison to control) with *p* < 0.05

Our results demonstrate a dose-dependent effect of rESW on gene expression of muscle-specific genes (Fig. 5). After single exposure, Pax7 is significantly up-regulated in the group with the highest air pressure (AP) and highest flux density (FD, A4) compared to the non-treated control. Interestingly, in the group with lower AP and FD, gene expression is down-regulated. After double exposure medium, AP and FD (B2 and B3) demonstrate the highest effect on primary muscle cells. When comparing single with double exposure, we see a significant up-regulation of Pax7 gene expression in the groups A3 and B3 (3 bar, 0.14 mJ/mm^2^) and a significant down-regulation between the groups A4 and B4 (4 bar, 0.19 mJ/mm^2^). MyoD demonstrates a gene expression pattern comparable to Pax7. Concerning gene expression of NCAM, our results demonstrate a significant up-regulation compared to the non-treated control group after single and double exposure in the groups A3 and B3 (3 bar, 0.14 mJ/mm^2^) and single exposure in group A4 (4 bar, 0.19 mJ/mm^2^). Double exposure with the highest EFD results in a significant down-regulation when compared to single exposure. Myf5 is significantly up-regulated after single exposure with the highest EFD (A4, 4 bar, 0.19 mJ/mm^2^) and after double exposure with the three highest EFD (groups B2–B4). However, whereas double exposure with the second highest EDF (3 bar, 0.14 mJ/mm^2^) results in the top value of Myf5 gene expression, double exposure with the highest EFD results in a significant down-regulation when compared to single exposure and group B3.

## Discussion

ESWT has become an established non-invasive procedure for the treatment of various musculoskeletal disorders over the last two decades [6]. Stimulating effects of ESWs on osteoblasts and fibroblasts were reported, as well as induction of neovascularization and increased expression of bone morphogenic proteins (BMPs) [28–32]. Beside the beneficial effect on tissue healing, ESWs have anti-inflammatory and analgesic effects [33, 34]. Furthermore, ESWT provides clinically favorable outcome to patients with breast cancer-related lymphedema [35]. Although there are some findings that indicate an acceleration of tissue healing, the biological mechanisms of ESWT causing these noticed effects have remained largely unknown.

Skeletal muscle injuries are the most common reason for missed playing time in athletes [1, 36]. The treatment modalities of structural muscle injuries are still limited. Recent clinical studies reported that ESWT can reduce pain and muscle tone in multiple sclerosis (MS) patients without adverse effects [37]. It is a therapeutic option in rehabilitative medicine to treat pain and muscle hypertonia in patients with cerebral palsy and stroke [16] and may modulate the rheology of muscles [38] as well as neurotransmission at the neuromuscular junction [39].

The aim of the present study was to investigate biological effects of rESWs on human primary skeletal muscle cells in vitro after single and double exposure with different EFDs. Both single exposure as well as double exposure influenced viability of the cells. Medium EFD (2 and 3 bar; 0.09 and 0.14 mJ/mm^2^) demonstrated the highest viability of muscle cells. Higher EFD resulted in low viability of the cells (Fig. 4). Although a tendency is clearly noticeable, the differences are not statistically significant. Moreover, it could be speculated that double exposure of shock waves with 4 bar and 0.19 mJ/mm^2^ which resulted in inhibition of cell viability could be due to mechanical cell destruction of these high EFD. These results are confirmed by gene expression analyses (see below). Summarizing the effects of shock wave application on muscle-specific gene expression, our results reveal that a significant increase was observed for all tested genes after single exposure to the highest EFD (4 bar, 0.19 mJ/mm^2^) and after double exposure with the medium EFDs (2 and 3 bar; 0.09 and 0.14 mJ/mm^2^, respectively). Double exposure of the highest EFD, however, results in a significant down-regulation when compared to single exposure with the same EFD. As this is in accordance to the viability results, it should be considered for therapeutical approaches; depending on the question and injury, more frequent applications with lower EFDs could be better than a single application with a higher EFD. However, this has to be further analyzed in an in vivo model of muscle injuries.

Myf5 is a direct target gene of Pax7 and transcription of Myf5 directly varies with Pax7 levels. The fact that it has been shown in the literature that Myf5 directly varies with Pax7 does not necessarily mean that both genes need to be up- or down-regulated at the same degree. The gene expression of Pax7 in general is lower than of Myf5 as Pax7 is a hallmark protein of quiescent satellite cells. As we isolated cells from muscle biopsies, the percentage of Pax7 positive cells hallmarking satellite cells was comparatively low as expected.

Human skeletal muscle cell cultures were positive for Pax7 (Fig. 3a–c), NCAM (Fig. 3d–f), and Myf5 (Fig. 3g–i) demonstrated by immunofluorescence staining indicating the presence of a mixed muscle cell culture at an early time point of differentiation. Myosin staining (Fig. 3j–l) evidenced the presence of myotube formation of mature muscle cells. In our experiments, we worked with mixed cultures and according to the expression of Myf5 with cells at early stages of differentiation.

Previously, it has been reported that ESWT stimulates and accelerates regenerative processes of acute muscle injuries in adult Sprague-Dawley rats [40]. In this study, an acute cardiotoxin-induced injury was set into the quadriceps femoris muscle of rats in the experimental groups and analyzed by histomorphometry and immunohistochemistry.

Accordingly, rESWT might influence gene expression and cell division of satellite cells in human skeletal muscle in vivo. In general, these data support previous findings that ESWT has an influence on biological cell function. It is critical to note that in this mentioned study [40], muscle injury of the quadriceps femoris muscle was initiated using a cardiotoxin, causing irreversible depolarization until muscle fibers die. However, this model may be of only limited relevance to muscle injuries in sports medicine. It was recently found that in models of muscle injury, the nature of the injury model should be chosen carefully depending on the experimental design and desired outcome [41]. This is due to the fact that the trajectories of regenerative processes leading to regeneration may considerably vary among models [41, 42]. Accordingly, it appears necessary to repeat the experiments performed by Zissler et al. using a more relevant animal model of sports-related muscle injury [43, 44]. Moreover, Vetrano et al. reported promotion of cell growth of primary human tenocytes after exposure to fESWs in vitro. Because proliferation of tenocytes is considered to be crucial for tissue repair, these results suggest that the clinical benefits and the timing of ESWT may be explained by an increased effectiveness of tissue healing mechanisms [45]. Furthermore, exposure of human tenocytes to ESWs in vitro was able to induce cell proliferation and synthesis of type I collagen, both appearing to be more pronounced after 8 to 12 days following exposure [46]. Similar results were recently reported for human fetal foreskin fibroblasts after exposure to rESWs in vitro [47].

The EFD values of the rESWs reported in the present study may appear very small compared to EFD values of rESWs reported in clinical studies [6]. This is due to the fact that the EFD values reported in the present study are those measured at a distance of 13 mm to the applicator of the rESW device in water, whereas in clinical studies on rESWT, usually the EFD values obtained at the tip of the applicator are reported. The latter is due to the fact that unlike in the in vitro situation, the EFD of ESWs cannot be reliably determined in vivo. However, preliminary data indicated that for rESWs, measurements in muscle tissue and water are consistent [48].

Finally, it should be pointed out that a certain limitation of all studies revealing enhanced cell viability and gene expression in connective tissue cells after exposure to ESWs is its in vitro nature. Working with primary human cells instead of cell lines has some disadvantages as numerous factors could affect gene expression. Different age of the donors is not the only factor in vivo that might influence gene expression: gender, medications, traumatic vs. degenerative disorders are just a few of them. The aim of our study was to look for general regulating factors under the influence of rESWs irrespective of age or gender. Accordingly, the results of these studies cannot directly be translated to the in vivo situation. On the other hand, results of in vitro studies are very useful for stimulating novel research in vivo. In this regard, the results of the present study, which to our knowledge represent the first biological effects of rESWs on human primary skeletal muscle cells, may serve as a starting point for future studies in vivo to evaluate the significance and relevance of rESWT on the process of muscle healing*.*

## Conclusion

In this in vitro study, we isolated and cultured primary human skeletal muscle cells from biopsy specimens and investigated the effect of radial extracorporal shock wave therapy (rESWT) on human skeletal muscle cells. To the best of our knowledge, this is the first study to provide evidence that rESWT has the potential to modulate the biological function of primary human muscle cells. rESWT influences human skeletal muscle cell viability and regulates the gene expression of muscle cell proteins in vitro, whereas in our study, rESWT had a higher impact on cell differentiation than on cell viability. rESWT could therefore offer promising options in the therapy of sports-related structural muscle injuries in Sports Medicine.

## Abbreviations

EFD: Energy flux density
ESWT: Extracorporeal shock wave therapy
fESWs: Focused extracorporeal shock waves
MPa: Megapascals
MRF: Myogenic regulatory factor
MS: Multiple sclerosis
Myf5: Myogenic factor 5
MyoD: Myogenic differentiation
NCAM: Neural cell adhesion molecule
Pax7: Paired box protein 7
PRP: Platelet rich plasma
rESWs: Radial extracorporeal shock waves

## References

[1] Ekstrand J, Hagglund M, Walden M (2011). Epidemiology of muscle injuries in professional football (soccer). Am J Sports Med.

[2] Reurink G, Goudswaard GJ, Tol JL, Verhaar JA, Weir A, Moen MH (2012). Therapeutic interventions for acute hamstring injuries: a systematic review. Br J Sports Med.

[3] Reurink G, Goudswaard GJ, Moen MH, Weir A, Verhaar JA, Bierma-Zeinstra SM, et al. Rationale, secondary outcome scores and 1-year follow-up of a randomised trial of platelet-rich plasma injections in acute hamstring muscle injury: the Dutch Hamstring Injection Therapy study. Br J Sports Med. 2015;49(18):1206–12. 10.1136/bjsports-2014-094250. Epub 2015 May 4. 10.1136/bjsports-2014-094250 25940636

[4] Reurink G, Goudswaard GJ, Moen MH, Weir A, Verhaar JA, Tol JL (2014). Myotoxicity of injections for acute muscle injuries: a systematic review. Sports Med.

[5] Hamid MS, Yusof A, Mohamed Ali MR (2014). Platelet-rich plasma (PRP) for acute muscle injury: a systematic review. PLoS One.

[6] Schmitz C, Csaszar NB, Milz S, Schieker M, Maffulli N, Rompe JD (2015). Efficacy and safety of extracorporeal shock wave therapy for orthopedic conditions: a systematic review on studies listed in the PEDro database. Br Med Bull.

[7] Rompe JD, Furia J, Weil L, Maffulli N (2007). Shock wave therapy for chronic plantar fasciopathy. Br Med Bull.

[8] Csaszar NB, Angstman NB, Milz S, Sprecher CM, Kobel P, Farhat M (2015). Radial shock wave devices generate cavitation. PLoS One.

[9] Schmitz C, Csaszar NB, Rompe JD, Chaves H, Furia JP (2013). Treatment of chronic plantar fasciopathy with extracorporeal shock waves (review). J Orthop Surg Res.

[10] Ogden JA, Toth-Kischkat A, Schultheiss R. Principles of shock wave therapy. Clin Orthop Relat Res. 2001;(387):8–17. 10.1097/00003086-200106000-00003 11400898

[11] Cacchio A, Rompe JD, Furia JP, Susi P, Santilli V, De Paulis F (2011). Shockwave therapy for the treatment of chronic proximal hamstring tendinopathy in professional athletes. Am J Sports Med.

[12] Speed CA (2004). Extracorporeal shock-wave therapy in the management of chronic soft-tissue conditions. J Bone Joint Surg Br.

[13] Speed CA, Nichols D, Richards C, Humphreys H, Wies JT, Burnet S (2002). Extracorporeal shock wave therapy for lateral epicondylitis—a double blind randomised controlled trial. J Orthop Res.

[14] Speed CA, Nichols D, Wies J, Humphreys H, Richards C, Burnet S (2003). Extracorporeal shock wave therapy for plantar fasciitis. A double blind randomised controlled trial. J Orthop Res.

[15] Speed CA, Richards C, Nichols D, Burnet S, Wies JT, Humphreys H (2002). Extracorporeal shock-wave therapy for tendonitis of the rotator cuff. A double-blind, randomised, controlled trial. J Bone Joint Surg Br.

[16] Wang T, Du L, Shan L, Dong H, Feng J, Kiessling MC (2016). A prospective case-control study of radial extracorporeal shock wave therapy for spastic plantar flexor muscles in very young children with cerebral palsy. Medicine (Baltimore).

[17] Ramon S, Gleitz M, Hernandez L, Romero LD (2015). Update on the efficacy of extracorporeal shockwave treatment for myofascial pain syndrome and fibromyalgia. Int J Surg.

[18] Manganotti P, Amelio E (2005). Long-term effect of shock wave therapy on upper limb hypertonia in patients affected by stroke. Stroke.

[19] Hawke TJ, Garry DJ (2001). Myogenic satellite cells: physiology to molecular biology. J Appl Physiol (1985).

[20] Schultz E, Chamberlain C, McCormick KM, Mozdziak PE (2006). Satellite cells express distinct patterns of myogenic proteins in immature skeletal muscle. Dev Dyn.

[21] von Maltzahn J, Jones AE, Parks RJ, Rudnicki MA (2013). Pax7 is critical for the normal function of satellite cells in adult skeletal muscle. Proc Natl Acad Sci U S A.

[22] Zammit PS, Heslop L, Hudon V, Rosenblatt JD, Tajbakhsh S, Buckingham ME (2002). Kinetics of myoblast proliferation show that resident satellite cells are competent to fully regenerate skeletal muscle fibers. Exp Cell Res.

[23] Tajbakhsh S, Buckingham M (2000). The birth of muscle progenitor cells in the mouse: spatiotemporal considerations. Curr Top Dev Biol.

[24] Seale P, Sabourin LA, Girgis-Gabardo A, Mansouri A, Gruss P, Rudnicki MA (2000). Pax7 is required for the specification of myogenic satellite cells. Cell.

[25] Zammit PS, Relaix F, Nagata Y, Ruiz AP, Collins CA, Partridge TA (2006). Pax7 and myogenic progression in skeletal muscle satellite cells. J Cell Sci.

[26] McKinnell IW, Ishibashi J, Le Grand F, Punch VG, Addicks GC, Greenblatt JF (2008). Pax7 activates myogenic genes by recruitment of a histone methyltransferase complex. Nat Cell Biol.

[27] Stewart JD, Masi TL, Cumming AE, Molnar GM, Wentworth BM, Sampath K (2003). Characterization of proliferating human skeletal muscle-derived cells in vitro: differential modulation of myoblast markers by TGF-beta2. J Cell Physiol.

[28] Wang CJ, Wang FS, Yang KD, Weng LH, Hsu CC, Huang CS (2003). Shock wave therapy induces neovascularization at the tendon-bone junction. A study in rabbits. J Orthop Res.

[29] Wang CJ (2003). An overview of shock wave therapy in musculoskeletal disorders. Chang Gung Med J.

[30] Hofmann A, Ritz U, Hessmann MH, Alini M, Rommens PM, Rompe JD (2008). Extracorporeal shock wave-mediated changes in proliferation, differentiation, and gene expression of human osteoblasts. J Trauma.

[31] Frairia R, Berta L (2011). Biological effects of extracorporeal shock waves on fibroblasts. A review. Muscles Ligaments Tendons J.

[32] Contaldo C, Hogger DC, Khorrami Borozadi M, Stotz M, Platz U, Forster N (2012). Radial pressure waves mediate apoptosis and functional angiogenesis during wound repair in ApoE deficient mice. Microvasc Res.

[33] Mariotto S, de Prati AC, Cavalieri E, Amelio E, Marlinghaus E, Suzuki H (2009). Extracorporeal shock wave therapy in inflammatory diseases: molecular mechanism that triggers anti-inflammatory action. Curr Med Chem.

[34] Maier M, Averbeck B, Milz S, Refior HJ, Schmitz C. Substance P and prostaglandin E2 release after shock wave application to the rabbit femur. Clin Orthop Relat Res. 2003;(406):237–45. 10.1097/01.blo.0000030173.56585.8f 12579024

[35] Bae H, Kim HJ (2013). Clinical outcomes of extracorporeal shock wave therapy in patients with secondary lymphedema: a pilot study. Ann Rehabil Med.

[36] Ekstrand J, Hagglund M, Walden M (2011). Injury incidence and injury patterns in professional football: the UEFA injury study. Br J Sports Med.

[37] Marinelli L, Mori L, Solaro C, Uccelli A, Pelosin E, Curra A (2015). Effect of radial shock wave therapy on pain and muscle hypertonia: a double-blind study in patients with multiple sclerosis. Mult Scler.

[38] Kisch T, Wuerfel W, Forstmeier V, Liodaki E, Stang FH, Knobloch K (2016). Repetitive shock wave therapy improves muscular microcirculation. J Surg Res.

[39] Kenmoku T, Ochiai N, Ohtori S, Saisu T, Sasho T, Nakagawa K (2012). Degeneration and recovery of the neuromuscular junction after application of extracorporeal shock wave therapy. J Orthop Res.

[40] Zissler A, Steinbacher P, Zimmermann R, Pittner S, Stoiber W, Bathke AC (2017). Extracorporeal shock wave therapy accelerates regeneration after acute skeletal muscle injury. Am J Sports Med.

[41] Hardy D, Besnard A, Latil M, Jouvion G, Briand D, Thepenier C (2016). Comparative study of injury models for studying muscle regeneration in mice. PLoS One.

[42] Mahdy MA, Lei HY, Wakamatsu J, Hosaka YZ, Nishimura T (2015). Comparative study of muscle regeneration following cardiotoxin and glycerol injury. Ann Anat.

[43] Hasselman CT, Best TM, Seaber AV, Garrett WE (1995). A threshold and continuum of injury during active stretch of rabbit skeletal muscle. Am J Sports Med.

[44] Nikolaou PK, Macdonald BL, Glisson RR, Seaber AV, Garrett WE (1987). Biomechanical and histological evaluation of muscle after controlled strain injury. Am J Sports Med.

[45] Vetrano M, d'Alessandro F, Torrisi MR, Ferretti A, Vulpiani MC, Visco V (2011). Extracorporeal shock wave therapy promotes cell proliferation and collagen synthesis of primary cultured human tenocytes. Knee Surg Sports Traumatol Arthrosc.

[46] Leone L, Vetrano M, Ranieri D, Raffa S, Vulpiani MC, Ferretti A (2012). Extracorporeal shock wave treatment (ESWT) improves in vitro functional activities of ruptured human tendon-derived tenocytes. PLoS One.

[47] Hochstrasser T, Frank HG, Schmitz C (2016). Dose-dependent and cell type-specific cell death and proliferation following in vitro exposure to radial extracorporeal shock waves. Sci Rep.

[48] Kearney CJ, Prevost T, Socrate S, Cleveland RO, Spector M (2010). Pressure-time profiles of a focused and a radial shockwave device: measurements in tissue, ex vivo, and in a water bath. J Acoust Soc Am.

